# Guilty or not guilty by reason of insanity? a comparative study of murderers referred for psychiatric examination by court order

**DOI:** 10.1186/s40352-023-00230-z

**Published:** 2023-09-05

**Authors:** Anat Yaron Antar

**Affiliations:** Department of Criminology, Max Stern Yezreel Valley College, Jezreel Valley, Israel

**Keywords:** Murder, Insanity, Mental disorders, Schizophrenia, Forensic psychiatry, Forensic characteristics

## Abstract

**Background:**

Some murders are committed under the influence of a psychotic state resulting from a mental disorder, mainly schizophrenia. According to the law in many countries, people with mental disorders do not have criminal responsibility. They are defined as not guilty due to insanity (insanity defense) and therefore cannot be punished. In Israel, in recent years, more lawyers are requesting psychiatric opinions for the murder defendants they represent. This study aims to explore the differences between two groups of murderers: individuals who committed murder and were found not guilty by reason of insanity (NGRI) and individuals who committed murder and were found responsible and guilty. The comparison is made from a broad perspective by examining sociodemographic factors and psychiatric factors as well as criminological and forensic factors.

**Methods:**

This study, conducted in Israel, analyzes the sociodemographic and forensic differences between 72 individuals who committed murder and were found not guilty by reason of insanity (NGRI) and 56 individuals who committed murder and were found responsible for their actions and fit to stand trial (guilty).

**Results:**

The findings show that NGRI participants were more likely to be from central areas, to be Jewish (rather than Arab), to be diagnosed with schizophrenia and have a background of hospitalizations before committing the murder, to have remained at the murder scene and/or called for help, and to be less likely to have committed the murder with a partner.

**Conclusions:**

The study’s findings are explained and the limitations discussed. The findings add to the existing knowledge base about murder by reason of insanity and the differences between NGRI and criminal murderers. The characteristics of the NGRI group found here can help to identify risk groups and to develop and implement prevention programs for people with mental disorders who are at risk of violent behavior.

## Background

Murder as a crime with the most devastating consequences, namely, the taking of human life, which carries the most severe punishment, has been the focus of much research (e.g., Crabbe et al., 2008; Flynn et al., 2021; James and Proulx, 2016; Richard-Devantoy et al., 2009a, b; Santtila et al., 2003; Whiting et al., 2022). Although murder is, by definition, an act committed with malicious intent, some acts of murder are committed out of insanity by individuals suffering from psychotic mental disorders (Fazel et al., 2009; Peled et al., 2001). According to the law in many countries, these individuals do not have criminal responsibility. They are defined as not guilty due to insanity (insanity defense) and therefore cannot be punished; instead, they are involuntarily hospitalized in psychiatric wards. The possibility that individuals are not punished for murders they committed raises the need for a better understanding of the characteristics of murder under the influence of a psychotic episode (insanity) and a clearer distinction between the characteristics of these murders and murderers and others.

A plethora of studies in the fields of forensic psychiatry and criminology have examined the topic of murder and insanity (Crabbe et al., 2008; Fazel et al., 2014; Golenkov et al., 2011): the former focused almost exclusively on the sociodemographic and psychiatric characteristics of the murderers (Martone et al., 2013), whereas the latter concentrated on serial or sexual murderers (e.g., James and Proulx, 2016; Keppel and Walter, 1999).

There is, however, a dearth of studies examining the criminological aspects of not guilty by reason of insanity (NGRI) murderers (e.g., the behavior of the assassin at the crime scene) or comparing NGRI and guilty murderers (i.e., responsible for the crime) and the murders they commit. Therefore, the aim of this study, which was conducted in Israel, is to fill this lacuna by exploring the characteristics of murderers and murder cases and analyzing and clarifying the differences between NGRI and other murderers (who are not insane). Its specific objective is to compare both the background characteristics (sociodemographic, criminological, and psychiatric) of these two groups of murderers and their forensic characteristics as presented by their behavior at the crime scene.

### Murder among the mentally ill

Studies from the last two decades have reported a significant correlation between mental disorders, mainly psychotic disorders, and tendencies toward violent behavior (Fazel et al., 2009, 2014; Hachtel et al., 2021; Swinson et al., 2011; Whiting et al., 2022). Most individuals with mental disorders who were involved in violent crimes were diagnosed with schizophrenia. Their violent acts generally took place during a psychotic episode in which they felt threatened by delusions and hallucinations, which are the main psychotic characteristics associated with violence (Peled et al., 2001). Kim (2019) examined the crime prevalence in Korea among individuals with schizophrenia compared to the general population. She found that while the crime rates among individuals with schizophrenia were significantly lower than among the general population in most types of crimes, including violent crimes other than murder, intellectual crimes, and theft, they were significantly higher in murder (about five times), arson (six times), and drug-related crimes (two times). These data are significantly higher than the prevalence of schizophrenia among the general population (American Psychiatric Association, 2013).

Various studies have found that the risk of committing violent crime or murder is greater among individuals with psychosis (with and without comorbid substance abuse) than among the general population (Lindquist & Allebeck, 1990; Brennan et al., 2000; Fazel et al., 2009; Flynn et al., 2021; Short et al., 2013; Soyka et al., 2004; Tikasz et al., 2016). In a clinical survey examining the rate of mental disturbances among individuals convicted of murder in England and Wales, Shaw et al. (2006) noted that 5% of the convicted murderers were diagnosed with schizophrenia. Bauer et al. (2009) also reported that 5% of violent psychiatric patients attempted murder.

Although most studies found a correlation between schizophrenia and violent behavior (including murder), it should be emphasized that most patients with mental disorders, including psychotic disorders, are not violent. These studies also examined the mediating factors between schizophrenia and violence, specifically substance abuse comorbidity (Elbogen & Johnson, 2009; Fazel et al., 2009; Spidel et al., 2010).

### The legal aspect of insanity

Insanity is an issue which straddles two different fields of science: medicine and law. The legal system comprises two general requirements for criminal sanctions: *mens rea*, the intention to commit a crime, and *actus reus*, the actual crime committed and related behaviors. The insanity defense, which exists in the criminal law of many countries, refers to the belief that certain mental disorders or states can affect the individual’s ability to form *mens rea* and understand the wrongness of their action (Feurstein et al., 2005). In other words, the law also recognizes that there are situations in which a person may commit an offense under the influence of a mental disorder, meaning an illness that impairs a person’s judgment and reality testing, i.e., psychotic disorders.

### The insanity defense in Israel

The insanity defense exists in the Israeli Penal Law (1977) Article 34 H Insanity (Amendment 57) which states:

No person shall bear criminal responsibility for an act committed by them, if – at the time the act was committed, because of an illness that adversely affected their spirit or because of a mental impediment – they lacked any real ability – (1) to understand what they did or the wrongful nature of their act; or (2) to abstain from committing the act.

According to the law, not every offense by a person with a mental disorder is necessarily committed out of insanity and exempts them from criminal liability. Edna Arbel (who served as a judge on Israel’s Supreme Court 2004–2014) clarified in one of her rulings on the case of a woman with mental disorder accused of abusing and assaulting her children (Criminal Appeal 10,166/09) that there must be a causal connection between the mental illness or disability and the inability to understand the act or abstain from it.

A psychiatric opinion is not written in every murder case. In accordance with the insanity defense, after suspects of murder (or any other crime) have been investigated and an indictment has been filed against them, the judge can decide whether to refer them to an ambulatory psychiatric examination or psychiatric observation in a psychiatric hospital. Usually the court decides to refer a murderer to a psychiatric examination if the defendant has a previous psychiatric background, the law enforcement officials (e.g., judge, police, prison service, defense attorney) notice strange behavior, or the defense attorney requests it as part of the line of defense or reasoning or to convince the judge. At the end of the psychiatric observation process, the psychiatrist writes an opinion regarding criminal responsibility and the ability to stand trial. The judge uses this opinion to decide whether to continue the criminal proceedings or whether to stop them on the basis that the person is not guilty due to insanity. In the latter case, a court order will refer to the person to a psychiatric hospital, according to Sect. 15(b) of the Mental Health Care Act 1991:

[In the case of] a defendant who was charged with criminal prosecution and the court found that they had committed the offense of which they were charged but decided, on the basis of evidence presented by one of the litigants or evidence brought at its own initiative, that the defendant was ill at the time of the act and therefore not punishable and that the defendant is still ill, the court will order that the defendant is hospitalized or receives medical treatment.

Data received as part of a freedom of information request (a request for information that is not made public) show that from 2013 to 2018, between 104 and 121 murders were committed in Israel every year and between 60 and 74 indictments were filed (Israel Police, 2019). Recent years have seen a significant increase in murders: 136 murders in 2019, 138 in 2020, and 161 in 2021. Between 1989 and 2019, 111 cases were closed due to insanity. (The number of cases referred to psychiatric examination regarding sanity at the time of the murder is, however, unknown). It was not possible to obtain from the state institutions an orderly record of the number of indictments filed (not all murder cases are solved, so not all of them are charged) and the number of defendants referred for psychiatric examination.

### NGRI versus guilty murderers

A few studies have focused on the differences between murderers with mental disorders who were found NGRI and other murderers. One group of studies focused on sociodemographic and psychiatric characteristics. Richard-Devantoy et al. (2009a) examined the social, clinical, and forensic differences between 14 murderers with schizophrenia and 73 murderers with no psychiatric disorder and compared their relationships with their victims. The findings of their study revealed that the former were characterized by a specific socio-professional status (unmarried, living alone, and jobless), previous psychiatric history, and significantly more encounters with the police than the latter. Victims of murderers with schizophrenia were family members in 21% of cases, acquaintances in 57%, and strangers in 14%. It was found far more likely for the victim to be known to the perpetrator in the schizophrenia group than in the no mental disorder group. The researchers concluded that the main difference between the two groups is the psychopathology of the morbid process and the fact that murderers with schizophrenia were more likely to murder intimates than strangers.

Chen et al. (2018) analyzed a random sample of 20% of all archival records of murderers in China who were assessed by the West China Forensic Central Medical Service between 1998 and 2006 and found significant differences between murderers with schizophrenia and murderers with no psychotic disorder in, specifically, age, education, occupation, marital status, and relationship to their victim. Murderers with schizophrenia were found to be older, less educated, and more socially isolated than their non-psychotic counterparts. In addition, the estimated risk of reoffending was higher in the schizophrenia group than the non-psychotic group, even after controlling for demographic differences. Despite many of the murderers reporting long histories of mental disorders, about 40% of those with schizophrenia had never had any psychiatric treatment and less than 4% were in treatment at the time of the murder.

Another group of studies focused on the relationship between psychotic murderers and their victims. In a study conducted in Holland and Germany, Nijman et al. (2003). compared men with psychosis who committed assaults (not only murder) to men who committed assaults but were not found to be psychotic. They showed that in every case of serious assault, the men with psychosis knew their victims and thus concluded that it is very rare for individuals with psychosis to murder people unknown to them. Similarly, Baxter et al. (2001) and Slovenko (2003) indicated that cases of assault and murder within the family circle are relatively common among patients with schizophrenia. In fact, a meta-analysis of seven studies found that murdering a total stranger was extremely rare (Nielssen et al., 2009). These findings regarding acquaintance with the victim have been replicated in a number of other studies (Belli et al., 2010; Golenkov et al., 2011; Joyal et al., 2004; Richard-Devantoy et al., 2009a). In contrast, Leong and Silva (1995) found lower rates of murderers with schizophrenia who were acquainted with their victims (52%), and Chen et al. (2018) found that murderers diagnosed with schizophrenia in China had a greater tendency to attack strangers than their counterparts in western countries.

Only a few studies seem to have dealt with the criminal forensic characteristics of psychotic murder. Yaron Antar et al. (2020) found that 47% of NGRI murderers remained at the scene of the crime after committing the murder; however, they did not compare this finding to other murderers (not insanity). Mehdi et al. (2014) examined the pattern of criminal behavior of three groups of murderers: those with psychotic disorders, those with personality disorders, and those with no mental disorders. They found differences in the types of weapons used and the behaviors following the murders. Regarding the former, while those with no mental disorders usually used a lethal weapon to carry out the murder (such as a gun or knife), most of the murderers with psychotic disorders did not use a weapon (they set the victim on fire or strangled the victim). Regarding the latter, murderers with psychotic disorders, unlike non-psychotic murderers, tended not to leave the scene of the crime.

### The current study

While previous studies on NGRI murderers have focused mainly on sociodemographic and psychiatric characteristics, the current study explores the differences between these two groups from a broad perspective by examining sociodemographic factors and psychiatric factors as well as criminological and forensic factors. Such a comparison can, in my opinion, provide a better understanding of murder for reasons of insanity. This understanding can also be useful in dealing with the increase in the number of insanity defense claims in murder cases by preventing murderers who did not commit murder under the influence of a psychotic episode resulting from a mental disorder from using the insanity defense to escape punishment.

Based on the above literature review, this study proposed the following four hypotheses regarding differences between the study groups:

1. Differences will be found between the groups in sociodemographic characteristics. The NGRI group will comprise more unmarried people than the guilty group because people with schizophrenia are often more socially isolated, unmarried, and have limited social relationships outside of their family (American Psychiatric Association, 2013). The guilty group will comprise more Arab participants due to the significant increase in the number of criminal murders in Arab society in recent years: in 2020 and 2021 there was a 16.7% increase in the number of murder cases opened (138 and 161, respectively) (Central Bureau of Statistics, 2022a; Israel Police, 2022).
2. Differences will be found between the groups in psychiatric characteristics. The NGRI group will comprise more people with a diagnosis of schizophrenia and with more previous hospitalizations than the guilty group; as mentioned above, most individuals with mental disorders who were involved in violent crimes were diagnosed with schizophrenia.
3. Differences will be found between the groups in criminal background characteristics. The NGRI group will comprise fewer people with a criminal background than the guilty group because they commit their offenses under the influence of the disease and not as typical criminal behavior.
4. Differences will be found between the groups in murder-related characteristics. The NGRI group will show the following tendencies: less early planning due to an expressive and non-instrumental motive, a common motivation of strong fear, and impaired judgment; less use of weapons due to their reduced accessibility and less early planning; more murders of family members due to being the main characters with whom they interact; and a greater likelihood to remain at the murder scene than the guilty group due to their strong and absolute belief in the false thought which justifies their action.

## Method

### Participants

The maximum security unit at Sha’ar Menashe Mental Health Center is a national facility comprising four closed psychiatric wards with a maximum level of security. It is the only such institution in Israel and includes patients from all over the country who need maximum supervision. Some of those hospitalized in the unit are transferred from other hospitals due to violent behavior or attempted escape, and some are hospitalized due to a court order for hospitalization or a court order for psychiatric observation after committing offenses, usually severe violent offenses, including murder.

The current study examined the hospital records (investigation material, indictments, admission summaries, and expert testimonies) of all 128 inpatients who had committed murder and been hospitalized in the maximum security unit from its opening in 1997 until 2020. All of them had committed murder between 1982 and 2020 and undergone observation in a psychiatric institution (some in the maximum security division and some in another psychiatric hospital and, subsequently, hospitalized in the maximum security unit). The study compared the medical and criminal files of 72 individuals who committed murder and were found NGRI by the court which ordered their psychiatric hospitalization under Sect. 15 of the Treatment of the Mentally Ill Law, 1991 (hereafter, the NGRI group) with the files of 56 individuals who, according to psychiatric observation and the court’s decision, were found responsible for their actions and fit to stand trial (hereafter, the guilty group). It should be noted that the groups are not equal in size because all the murders whose perpetrators were hospitalized in the maximum security division were examined for this study and not just a selected sample.

The NGRI group included the files of all patients who were hospitalized in the maximum security unit from 1997 to 2020. It should be noted that these 72 patient files represent more than 60% of the overall population of NGRI murderers; according to information from the Israel Police (2019), 111 murder cases were closed between 1989 and 2019 on the grounds that the defendant was not punishable due to insanity. The guilty group included the files of all 56 individuals who arrived in the maximum security unit from 1997 to 2020 for psychiatric observation and were found fit to stand trial by both the psychiatric evaluation and the court. Files in which the opinions were inconsistent were not included in the study (a total of three files). Table 1 describes the characteristics of the research and control groups.

**Table 1:** Description of sociodemographic characteristics for the NGRI group and the guilty group

		NGRI Group (N = 72)		Guilty Group (N = 56)	
**Age (at time of murder)**		M = 35.01, SD = 12.91		M = 34.07, SD = 12.31	
**Country of origin**	Israel	42	58.3%	44	78.6%
	Former Soviet Union	16	22.2%	8	14.3%
	Ethiopia	5	6.9%	1	1.8%
	Other	9	12.5%	3	5.4%
**Religion**	Jewish	60	83.3%	26	46.4%
	Muslim	8	11.1%	27	48.2%
	Christian (Arab)	1	1.4%	-	-
	Christian	2	2.8%	-	-
	Druze	1	1.4%	1	1.8%
	Unknown	-	-	2	3.6%
**Marital status (at time of murder)**	Single	45	62.5%	25	44.6%
	Married	11	15.3%	21	37.5%
	Divorced	16	22.2%	10	17.9%
**Place of residence**	City	63	87.5%	39	69.6%
	Village	7	9.8%	16	28.6%
	Tourist	2	2.8%	-	-
	Other	-	-	1	1.8%
**Area of residence**	Center	41	56.9%	10	17.9%
	South	8	11.1%	5	8.9%
	North	21	29.2%	41	73.2%
	Tourist	2	2.8%	-	-

### Instruments and procedure

The study was approved by the IRB of Sha’ar Menashe Mental Health Center. It includes a retrospective examination of medical records. Confidentiality and anonymity of the records involved in the study was strictly maintained and, therefore, informed consent of patients whose files were evaluated was not required.

The documents included the expert psychiatric testimony submitted to the court, the indictment, and summaries of previous hospitalizations. Two research assistants collected the data from the medical files (physical and computerized) using a pre-prepared data collection form. All data were reexamined by the principal researcher. The following data were extracted from the medical files:

1. Sociodemographic variables (during the period preceding the murder): age, marital status, religion, country of birth, years since immigration to Israel, and residential area.
2. Psychiatric background: number of previous hospitalizations, age at first hospitalization, psychiatric diagnosis.
3. Criminal background: previous criminal offenses, age at first offense, types of prior offenses, number of incarcerations or court-ordered hospitalizations, drug use (presence of drug use and age when first used drugs).
4. Murder characteristics: variables relating to the murder – method of murder (shooting, stabbing, level of violence), degree of premeditation, declared reason for the murder, reason for the murder according to the indictment, number of people murdered, behavior following the murders; relationship between murderer and victim – degree of acquaintance with the victim, previous confrontations.

### Statistical analysis

In order to compare the groups, chi-square analyses were used for categorical variables and independent samples t-tests were used for continuous variables. Bonferroni correction was applied to account for multiple comparisons (α < 0.0025). After identifying variables that differentiate between the groups, hierarchical logistic regression was computed to obtain a more complete picture and to identify which of the variables contribute to the NGRI and guilty classification. All significant variables were entered as predictors.

## Results

### Preliminary analysis

Differences between the NGRI group and the guilty group were examined according to the following topics (see Table 2).

**Table 2:** Description of sociodemographic, psychiatric, and criminal background and murder characteristics of NGRI and guilty groups

	All Sample (N=128)*	Guilty Group (n = 56)	NGRI Group (n = 72)	Statistic
**Demographics**				
Age at offence	34.9 (12.3)	34.1 (12.3)	35.6 (12.3)	t(126) = 0.48, p = 0.48
Migrant				χ^2^(1) = 5.85, p = 0.016
Yes	32.8% (42)	21.4% (12)	41.7% (30)	
No	67.2% (86)	78.6% (44)	58.3% (42)	
Family status				χ^2^(2) = 8.35, p = 0.015
Single	54.7% (70)	44.6% (25)	62.5% (45)	
Married	25.0% (32)	37.5% (21)	15.3% (11)	
Divorced	20.3% (26)	17.9% (10)	22.2% (16)	
Area of residence				χ^2^(1) = 21.41, p<0.001
Center	40.5% (51)	17.9% (10)	58.6% (41)	
Periphery	59.5% (75)	82.1% (46)	41.4% (29)	
Religion				χ^2^(1) = 22.24, p<0.001
Jewish	71.1% (86)	49.1% (26)	88.2% (60)	
Muslim	28.9% (35)	50.9% (27)	11.8% (8)	
**Psychiatric background**				
Hospitalization				χ^2^(1) = 26.48, p<0.001
Yes	50.8% (65)	25.0% (14)	70.8% (51)	
No	49.2% (63)	75.0% (42)	29.2% (21)	
Number of hospitalizations	3.6 (7.8)	1.1 (3.6)	5.5 (9.4)	t(95.4) = 3.65, p<0.001
**Criminal background**				
Criminal background				χ^2^(1) = 1.41, p = 0.24
Yes	62.0% (75)	67.9% (36)	57.4% (39)	
No	38.0% (46)	32.1% (17)	42.6% (29)	
Alcohol use				χ^2^(1) = 0.63, p = 0.43
Yes	44.7% (51)	49.0% (24)	41.5% (27)	
No	55.3% (63)	51.0% (25)	58.5% (38)	
Drug use				χ^2^(1) = 0.10, p = 0.76
Yes	54.4% (62)	52.8% (28)	55.7% (34)	
No	45.6% (52)	47.2% (25)	44.3% (27)	
**Murder characteristics**				
Place of murder				χ^2^(2) = 2.12, p = 0.35
Home	59.4% (76)	53.6% (30)	63.9% (46)	
Street	16.4% (21)	21.4% (12)	12.5% (9)	
Other	24.2% (31)	25.0% (14)	23.6% (17)	
Accomplice				χ^2^(1) = 12.35, p<0.001
Yes	9.4% (12)	19.6% (11)	1.4% (1)	
No	90.6% (116)	80.4% (45)	98.6% (71)	
Victim number				χ^2^(1) = 0.19, p = 0.67
One	90.6% (115)	89.3% (50)	91.5% (65)	
Two or more	9.4% (12)	10.7% (6)	8.5% (6)	
Victim acquaintance				χ^2^(1) = 0.41, p = 0.52
Yes	89.1% (114)	91.1% (51)	87.5% (63)	
No	10.9% (14)	8.9% (5)	12.5% (9)	
Victim relation				χ^2^(4) = 5.03, p = 0.28
Parents	19.5% (25)	12.5% (7)	25.0% (18)	
Spouse	21.9% (28)	26.8% (15)	18.1% (13)	
Family	15.6% (20)	14.3% (8)	16.7% (12)	
Acquainted	32.0% (41)	37.5% (21)	27.8% (20)	
Stranger	10.9% (14)	8.9% (5)	12.5% (9)	
Conflict				χ^2^(1) = 0.14, p = 0.71
Yes	68.0% (83)	69.8% (37)	66.7% (46)	
No	32.0% (39)	30.2% (16)	33.3% (23)	
Planning				χ^2^(1) = 0.37, p = 0.54
Yes	48.8% (60)	51.8% (29)	46.3% (31)	
No	51.2% (63)	48.2% (27)	53.7% (36)	
Weapon				χ^2^(3) = 3.40, p = 0.094
Gun	12.5% (16)	16.1% (9)	9.7% (7)	
Knife	51.6% (66)	58.9% (33)	45.8% (33)	
Beating with hands or object	18.8% (24)	16.1% (9)	20.8% (15)	
More than one	17.2% (22)	8.9% (5)	23.6% (17)	
Behavior after murder				χ^2^(3) = 36.20, p<0.001
Stay/help	42.0% (50)	13.2% (7)	65.2% (43)	
Leave/suppress evidence	45.4% (54)	62.3% (33)	31.8% (21)	
Suicide	4.2% (5)	9.4% (5)	0.0% (0)	
Other	8.4% (10)	15.1% (8)	3.0% (2)	
Injured victims				χ^2^(1) = 0.03, p = 0.86
Yes	17.2% (22)	17.9% (10)	16.7% (12)	
No	82.8% (106)	82.1% (46)	83.3% (60)	

* In some cases there were missing values such that the number does not always total 128 participants.

### Demographics characteristics

Significant differences were found in the following variables: area of residence [*χ*^*2*^(1) = 21.41, *p* < .001] and religion [*χ*^*2*^(1) = 22.24, *p* < .001]. As shown in Table 2, participants from Israel’s central residential area were more likely to be classified in the NGRI group, while participants from peripheral areas were more likely to be classified in the guilty group. Jewish participants were more likely to be classified in the NGRI group, while Muslim participants were more likely to be classified in the guilty group.

Differences close to significant (not significant due to Bonferroni correction) were found in the following variables: immigration [*χ*^*2*^(1) = 5.85, *p* = .016] and marital status [*χ*^*2*^(2) = 8.35, *p* = .015]. As shown in Table 2, the proportion of immigrants is higher in the NGRI group than the guilty group. Most of the murderers in both groups are unmarried, but the rate is higher in the NGRI group.

### Psychiatric background

Differences were found in the history of psychiatric hospitalization (yes or no) before the event [*χ*^*2*^(1) = 26.48, *p* < .001] and number of psychiatric hospitalizations [*t*(95.4) = 3.65, *p* < .001]. As shown in Table 2, participants with previous hospitalizations were more likely to be classified in the NGRI group and people classed as psychotic had a greater number of hospitalizations than the guilty group. In the NGRI group the large majority (88.9%) were diagnosed with schizophrenia; in the guilty group the majority (69.2%) had no psychiatric diagnosis, and only 11 participants (21.1%) were diagnosed with schizophrenia.

### Criminal background

No significant differences in criminal background were found.

### Murder characteristics

Significant differences were found regarding murders committed with accomplices [*χ*^*2*^(1) = 12.35, *p* < .001] and behavior at the scene after the murder [*χ*^*2*^(3) = 36.20, *p* < .001]. As shown in Table 2, in both groups most of the murders were committed alone; however, in the guilty group there were 11 murderers (19.6%) who committed the murder with an accomplice, while in the NGRI group there was only one such participant (1.4%). Participants who remained at the scene or called for help were more likely to be classified in the NGRI group; participants who left the scene, withheld evidence, or attempted suicide were more likely to be classified in the guilty group.

Not surprisingly, differences between the groups were also found in the motive for the murder (*χ*^*2*^ = 92.21, *p* < .001). In the NGRI group, the main motive was paranoid delusions (66.67%) which, in some cases, appeared in combination with hallucinations. In addition, among some of the participants, the delusions that led to the murder were delusions of envy (6.94%) or grandiosity (5.56%). The guilty group had various motives including revenge (30.36%), jealousy (12.5%), response to their wife’s intention to leave them (10.71%), economic problems (8.93%), previous conflicts (7.14%), terrorism (3.57%), or “family honor” (3.57%). In the remaining cases the motive was not described.

### NGRI group vs. guilty group – regression analysis findings

A hierarchical logistic regression was conducted to capture the variables that differentiate between the NGRI and guilty groups. Only the variables found significant in the first analyses, shown in Table 2, were inserted in the regression. The regression results are shown in Table 3, The directions of the differences between the groups are shown in Table 2 and were previously described in the preliminary analysis subsection.

In the first step of the regression analysis, demographic variables were entered including area of residence and religion. The first step of the model was significant, *χ*^*2*^(5, *N* = 112) = 37.03, *p* < .001], Cox and Snell *R*^*2*^ = 0.28, Nagelkerke *R*^*2*^ = 0.38, and correctly classified 73.2% of the cases. The second step of the model added information about previous hospitalizations and was significant, *χ*^*2*^(6, *N* = 112) = 49.12, *p* < .001 (step *χ*^*2*^(1) = 12.09, *p* = .001), Cox and Snell *R*^*2*^ = 36, Nagelkerke *R*^*2*^ = 0.48, and correctly classified 77.7% of the cases. The third step of the model added information about the murder incidence and was significant, *χ*^*2*^(9, *N* = 112) = 81.32, *p* < .001 (step *χ*^*2*^(3) = 32.20, *p* < .001), Cox and Snell *R*^*2*^ = 0.52, Nagelkerke *R*^*2*^ = 0.69, and correctly classified 85.7% of the cases.

Another interesting finding was that, after entering information about the murder characteristics, religion was no longer significant. A crosstabs analysis showed correlations between religion (only the two largest groups were examined: Jews and Muslims) and the two aforementioned variables associated with murder: accomplice [*χ*^*2*^(1) = 12.35, *p* < .001] and behavior at the murder scene [*χ*^*2*^(8) = 43.14, *p* < .001]. It was found that of the 12 participants (Jewish and Muslim) who committed murder with an accomplice, 11 were Muslims. In addition, 38 (63%) of the Jewish murderers remained at the scene and/or called for assistance compared to only 13% of the Muslim murderers. The implications of these findings are discussed in the following section.

**Table 3:** Hierarchical logistic regression predicting guilty/NGRI group (N = 112)*

		B	SE	Wald statistic	Significance	OR	95%CI
**Step 1**							
	(Constant)	1.34	0.67	4.04	0.045	3.83	
	Immigrant	0.28	0.52	0.30	0.59	1.33	0.48 − 3.70
	Single vs married	0.67	0.56	1.46	0.23	1.96	0.66-5.85
	Divorced vs married	0.01	0.70	0.00	0.99	1.01	0.26-3.95
	Periphery vs center	-1.69	0.50	11.65	0.001	0.18	0.07-0.49
	Muslim vs Jew	-1.67	0.54	9.50	0.002	0.19	0.07-0.54
**Step 2**							
	(Constant)	0.31	0.77	0.17	0.684	1.37	
	Immigrant	0.61	0.57	1.12	0.290	1.84	0.60-5.65
	Single vs married	0.77	0.60	1.66	0.198	2.15	0.67 − 6.90
	Divorced vs married	0.08	0.74	0.01	0.911	1.09	0.26-4.61
	Periphery vs center	-1.68	0.53	10.04	0.002	0.19	0.07-0.53
	Muslim vs Jew	-1.34	0.58	5.41	0.02	0.26	0.08-0.81
	Previous hospitalization	1.69	0.51	11.04	0.001	5.39	2.00-14.56
**Step 3**							
	(Constant)	2.15	1.07	4.04	0.044	8.60	
	Immigrant	0.95	0.74	1.66	0.198	2.60	0.61-11.09
	Single vs married	0.43	0.83	0.27	0.603	1.54	0.30-7.76
	Divorced vs married	0.07	0.91	0.01	0.936	1.08	0.18-6.44
	Periphery vs center	-1.92	0.70	7.55	0.006	0.15	0.04-0.58
	Muslim vs Jew	−0.97	0.72	1.82	0.178	0.38	0.09-1.55
	Previous hospitalization	2.02	0.65	9.70	0.002	7.55	2.12–26.92
	Accomplice	-2.58	1.28	4.04	0.045	0.08	0.01-0.94
	Leave scene vs stay	-2.23	0.70	10.13	0.001	0.11	0.03-0.42
	Suicide/other vs stay	-4.19	1.17	12.90	0.000	0.02	0.00-0.15

*The full sample was 128 participants; due to missing values only 112 participants remained.

## Discussion

The aim of this study was to examine the sociodemographic, psychiatric, criminal, and forensic differences between two groups of murderers: NGRI and guilty.

### Findings relating to demographic characteristics

The first finding regarding the demographic variables was that participants from Israel’s central residential area were more likely to be categorized as NGRI than those from peripheral areas. This finding is surprising as it could be expected that in the center of the country, where there tends to be better access to medical services, there will be fewer cases of murder under the influence of a psychotic disorder. Studies have indicated that individuals diagnosed with schizophrenia from rural and peripheral areas are significantly more likely to have committed a violent act than non-violent individuals with schizophrenia (Karabekiroğlu et al., 2016), and most of the repeat homicide offenses by individuals with schizophrenia were shown to be committed by people residing in rural areas with less access to psychiatric services (Golenkov et al., 2011).

One explanation for this may be the higher incidence of schizophrenia in urban areas (American Psychiatric Association, 2013) alongside the fact that most of the Israeli population lives in cities (73.9%) (Central Bureau of Statistics, 2022b); the big Israeli cities are in the center of the country, which therefore increases the statistical probability that more murderers will be from the central area. In addition, Israel is a small country and the peripheral areas are populated and include health services, unlike similar areas in other countries, such as Russia (Golenkov et al., 2011).

Another explanation for this finding may be related to the correlation found between the two variables of area of residence and religion. The crime rate (including violent crimes and murder) in the Arab (mostly Muslim) community in Israel tends to be higher than in the Jewish community (Israel Police, 2022). In addition, Arabs usually live in the country’s peripheral areas (Central Bureau of Statistics, 2008). The guilty group thus has a high percentage of peripheral residents as well as a high percentage of Arabs (relative to their proportion in the population). However, in the hierarchical logistic regression, after entering information on the murder characteristics, religion was found no longer significant while the area of residence remained significant.

The second finding regarding the demographic variables relates to the fact that Arabs participants were found more likely than Jewish participants to be categorized as guilty and not NGRI. If schizophrenia is relatively equally distributed in a population, no difference should be expected between the two study groups and the rate should correspond to population proportions (Arabs constitute about 21% of Israel’s total population [Central Bureau of Statistics in Israel, 2022b]). In reality, the proportion of Muslims in the guilty group was found to be higher than their proportion in the population and lower in the NGRI group than their proportion in the population.

Israel is a multicultural state with a Jewish majority and an Arab minority including Muslims, Christians, Druze, Circassians, and others. Life as an ethnic minority has implications for various aspects including crime. Many studies have suggested that crime rates are significantly higher among minorities than among the majority (Bierschbach & Bibas, 2016; Robinson and Williams, 2009; Walsh and Douglas, 2016). This is this case in Israel where the rates of arrests and convictions of suspects of Arab ethnic origin are significantly higher than those of the Jewish majority. Data from the last five years (2017–2021) indicate that most of the murders in Israel were committed by members of the Arab minority (Israel Police, 2022).

All of the above can explain the higher rate of Arabs in the guilty group relative to their proportion in the population. In addition, a crosstabs analysis showed that most of those who murdered with an accomplice were Arabs. This is perhaps related to the motive for the murder and the fact that honor killings or revenge murders are more common in Arab society (Chesler, 2010).

However, the reason for the lower incidence of Arabs in the NGRI group is less clear and requires a more in-depth examination. One possible explanation is the real difference between the groups: namely, there is less murder due to psychosis among the Arab population. However, in light of an absence of evidence of lower morbidity or greater response to psychiatric treatment and follow-up among minority populations in general and the Arab population in particular, this explanation sounds less plausible.

Another explanation relates to cultural differences. In recent years, cultural competence has become a popular term for a variety of strategies to address the challenge of cultural diversity in a range of fields including mental health services (Kirmayer, 2012). Despite the internationally accepted DSM and ICD classification standards, psychiatric diagnoses remain subjective and are based on the clinical impression of the examining psychiatrist. In Israel, most psychiatrists are Hebrew-speaking Jews born in Israel or in Russia and the Former Soviet Union (Yaron Antar & Einat, 2023). Therefore, psychiatric diagnoses are not always performed in the patient’s mother tongue, and the interpretation and conclusions are in the eyes of the examiner who is influenced, albeit unconsciously, by their own society and culture. As Israel is a multicultural country, further studies are needed to examine this issue in depth.

An additional explanation could be related to the issue of law enforcement among minorities. There is much theoretical and empirical literature in criminology and penology showing that minorities, relative to the general population, are discriminated against by the Criminal Justice System (Bierschbach & Bibas, 2016; Carson, 2015; Clair and Winter, 2016; Kennedy and Hansford, 2016; Klein, 2014; Uggen, 2016). This discrimination is manifested in policing, sentencing, and punishment (Baumgartner, 2016; Sklansky, 2017). In Israel, such discrimination has been documented regarding, in particular, the ethnic Arab minority (Fishman et al., 2006; Haklai, 2013; Hasisi & Weitzer, 2007; Shayo & Zussman, 2016). To the best of my knowledge, no studies have yet examined the role of discrimination in judicial decisions on insanity. It should be noted that both this and the previous explanation for the lower incidence of Arabs in the NGRI were not examined in the current study and are therefore suggested here with due caution. The findings of the present study suggest the need for more in-depth examination of the subject in future research.

Preliminary analyses also found close to significant differences in immigration and marital status variables: in the NGRI group, there is a high percentage of unmarried people relative to the guilty group and a high percentage of immigrants relative to their proportion in the population. These data support and strengthen other research findings indicating the high prevalence of immigrants among people with mental disorders who committed murder (Nielssen et al., 2011). Common explanations are associated either with the various unique social, cultural, and subcultural characteristics of immigrant populations (Bersani & Piquero, 2017) or with the numerous assimilation challenges, such as socialization processes, economic difficulties, and harsh living conditions (Kubrin & Mioduszewski, 2018). These explanations can explain delinquency in general and be relevant to both study groups. However, in the NGRI group there may be another explanation which relates to immigrants’ lack of knowledge regarding access to mental health services and the laws dealing with the possibility of involuntary medical care and hospitalization in cases of deterioration in the mental state of the family member. It can be assumed that this unfamiliarity prevented many mentally ill immigrants and their families from seeking psychiatric assistance, which resulted in a deterioration in their mental state and the execution of their violent criminal act. A more activist policy should therefore be implemented by various government ministries and health maintenance organizations. Such policies should incorporate, among others, identifying at-risk populations, organizing outreach activities, raising awareness among immigrant families, and providing information, such as hospitalization options, clinic and hospital locations, and essential phone numbers. This information should be easy to access and published on different platforms (e.g., electronic media, social networks such as Facebook, online newspapers, etc.) and in all the relevant languages.

### Findings relating to psychiatric background

As found in previous studies, in the current study too most of the murderers under the influence of psychosis were known to the psychiatric system (that is, they were hospitalized in the past) before committing the murder. The most common diagnosis in cases of murder under the influence of a psychotic disorder is schizophrenia.

### Findings relating to criminal background

No differences were found between the groups in the variables of substance abuse and criminal record. One explanation for this lack of difference might be that substance abuse is indeed a mediating factor between mental disorder and violence. Another explanation might be related to the lack of information as well as the lack of objective and official information regarding these data (Israeli law does not allow the transfer of information on a criminal background for the purpose of writing a psychiatric opinion or for research to external sources).

### Findings relating to the murder

The first major finding here concerns behavior at the scene of the crime after the murder. Murderers who remained at the scene and called for assistance were more likely to be classified as NGRI, while murderers who left the scene and/or concealed evidence were more likely to be classified as guilty. These findings are consistent with other studies on criminal (non-NGRI) murderers which indicated that criminal murderers flee the scene immediately after executing the murder and try to conceal the evidence (Balemba et al., 2014; Fujita et al., 2013).

A possible explanation for this type of behavior concerns NGRI offenders’ complete, absolute, and irrefutable belief in the authenticity of their delusions. Delusions are defined as false fixed ideas that are not shared by others and are not amenable to change in light of conflicting evidence (American Psychiatric Association, 2013; Yaron Antar et al., 2020). Persecutory or paranoid delusions are the belief that one is going to be harmed or harassed by an individual, an organization, or another group (American Psychiatric Association, 2013). Most criminal murderers (guilty group) perceive reality in a reasonable way (non-delusional) without damaging reality testing and thus commit murder from choice, aware that their act is illegal (Goodwill et al., 2014). However, most NGRI murderers are motivated by paranoid delusions, perceive reality in a psychotic and dangerous way (delusional reality) and, consequently, act in a deterministic and unavoidable manner. In other words, some NGRI murderers cannot distinguish between the prohibited and the permitted, while others understand that the act is prohibited but cannot avoid doing it.

According to this line of reasoning, Peled et al. (2001) maintained that the violent behavior of psychotic patients is a logical response to unrealistic thoughts (delusions) and false perceptions (hallucinations). Given that most of the study participants were diagnosed as suffering from persecutory delusions, it is reasonable to assume that their absolute belief that they would be harmed by the victim led them to act violently against them, knowing subjectively that they had done the right and unavoidable thing and that there was, therefore, no reason for them to hide by fleeing the murder scene or to conceal evidence.

The second finding relating to the murder concerns accomplices to the murder. In both the NGRI and guilty groups, most of the murders were committed alone without an accomplice. However, most of the murderers who committed the murder with an accomplice were found to be classified as guilty. This can be understood through the false perceptions (hallucinations) and unrealistic thoughts (delusions) of psychotic episodes; it is not shared by others but is their individual and separate reality (psychotic). The criminal motive, on the other hand, is rooted in reality and can be shared by several people and includes, for example, revenge, property, and murder for the honor of the family.

The third finding concerns differences in the murder motive. This finding is not surprising because it is the psychotic (mainly delusional) motive that turns the murder into a psychotic murder and leads to the decision that the perpetrator is not guilty by reason of insanity. In the NGRI group, the main motive was paranoid delusions which, in some cases, appeared in combination with hallucinations. As three of the murderers said in their psychiatric examination, “it was either me or him/them.” Paranoid delusions as a main motive is consistent with other studies (Provoost et al., 2022). In the guilty group a variety of motives were reported, primarily revenge, jealousy, response to their wife’s intention to leave them, and economic problems. In all these cases there was a reaction to real events.

Another important issue regarding behavior at the crime scene is related to the manner in which the murder was carried out. In some of the cases of the NGRI group, extreme cruelty was described during the execution of the murder, such as the dismemberment of the body after the murder (in several cases), taking of the victim’s head after the murder (in two cases), and murder by multiple stabbings (as many as 70 stabbings in one case). There were also descriptions of cases of murder by several extreme means, such as strangulation and then throwing the body from the balcony and beating with a heavy object and then strangulation. Such extreme cases were not found in the guilty group. This extreme cruelty could be explained as the result of strong fear stemming from complete belief in the delusion (false thought). For example, in cases of dismembering, the murderer explained that the threatening party (terrorist organization, an enemy mentioned in the Bible, etc.) would now not be able to use the body.

However, not all of the indictments were detailed enough to enable a thorough comparison between the groups. Follow-up studies should examine differences between the groups regarding behavior at the time of the murder.

### Limitations

The main limitation of this study concerns its reliance on missing information. Although the medical decisions were written in real time, they often lack significant details. There is, in addition, no uniformity in the reports: some are more detailed and some less. For example, some of the indictments and psychiatrist opinions do not include details about the offender’s behavior immediately following the murder, and some of the data concerning their criminal history and substance abuse were lacking, inaccurate, or dependent solely on self-reports. A more thorough examination of the cases at the time or access to additional databases (e.g., official police criminal records, history of follow-up in outpatient clinics, information from the family etc.) might improve their accuracy.

It is important to note that this study compared two groups of murderers who, having been sent for psychiatric observation, were either deemed fit to stand trial (guilty group) or deemed unfit (NGRI group). It did not include murderers who were sentenced with no need for psychiatric observation. However, if differences were found between these two groups, it can be cautiously assumed that additional and potentially more significant differences will be found between murderers from the NGRI group and murderers whose sanity was not in doubt.

## Conclusions

This study has added to the existing knowledge base about murder by reason of insanity and the differences between psychotic and criminal (non-psychotic) murder.

The characteristics of the NGRI group found here and the differences between the groups can help psychiatrists who write psychiatric opinions and judges to better understand the characteristics of murder by reason of insanity, especially with reference to the psychiatric background, the motive for the murder and the behavior at the scene after the murder. A better understanding of the distinction between the groups could lead to more accurate conclusions in psychiatric opinions, enabling people who need hospitalization and psychiatric treatment to receive it and preventing people who are responsible for their actions from escaping punishment.

The characteristics of the NGRI group found here and in previous studies can also help to identify risk groups and develop and implement prevention programs for people with mental disorders who are at risk of violent behavior, particularly people with a previous psychiatric background, a diagnosis of schizophrenia, drug use, and non-responsiveness to medical monitoring and medication. Practically, these findings can be used to propose that the Ministry of Health define a risk group and strengthen the continuum of care from hospitalization to the community for this group. Upon discharge, a psychiatrist should examine whether the individual is in a risk group, thus obliging the psychiatrist in the community mental health services to provide closer monitoring. This should include more frequent follow-up meetings and conversations with family members, informing them about the risk, how to identify a deteriorating mental state, how to contact emergency services, and more. Such surveillance treads a fine line between maintaining individual freedom and guaranteeing public safety and necessitates both determination and sensitivity. Identifying, defining, and focusing on risk groups is important in order to reduce and prevent the recurrence of such cases.

The scope of the phenomenon of murder due to a psychotic state resulting from schizophrenia highlights, once again, the need for emergency psychiatric services, which are not sufficiently developed in various countries including Israel. The contribution of these services may be expressed on two levels. On the first and primary level, they provide a response in crisis situations, prevent escalation, and can assist in referrals to treatment providers. On the second level, they reduce the criminalization of mentally challenged individuals, which is a direct result of family members contacting the police in the absence of other adequate responses.

Future studies should explore more fully characteristics that were missing in the medical files used to collect the data for this study: for example, data related to criminal background, substance abuse, and behavior at the scene of crime. In addition, Future studies might develop and deepen the research on the characteristics of murderers who act under the influence of a mental disorder. This will help to formulate a more complete and accurate definition of the risk group. Finally, I recommend future studies examining the question of bias or discrimination in psychiatric opinions and judicial decisions against minorities in Israel and elsewhere.

## Data Availability

The data that support the findings of this study are available but restrictions apply to the availability of these data, which were used under license for the current study, and so are not publicly available. Data are however available from the authors upon reasonable request and with permission of the IRB of Sha’ar Menashe Mental Health Center.
